# Development and Optimization of Erythromycin Loaded Transethosomes Cinnamon Oil Based Emulgel for Antimicrobial Efficiency

**DOI:** 10.3390/gels9020137

**Published:** 2023-02-06

**Authors:** Marwa H. Abdallah, Hanaa A. Elghamry, Nasrin E. Khalifa, Weam M. A. Khojali, El-Sayed Khafagy, Seham Shawky, Hemat El-Sayed El-Horany, Shaimaa El-Housiny

**Affiliations:** 1 Department of Pharmaceutics, College of Pharmacy, University of Ha’il, Ha’il 81442, Saudi Arabia; 2 Department of Pharmaceutics and Industrial Pharmacy, Faculty of Pharmacy, Zagazig University, Zagazig 44519, Egypt; 3 Department of Pharmaceutics, Faculty of Pharmacy, University of Khartoum, Khartoum 11115, Sudan; 4 Department of Pharmaceutical Chemistry, College of Pharmacy, University of Ha’il, Ha’il 81442, Saudi Arabia; 5 Department of Pharmaceutical Chemistry, Faculty of Pharmacy, Omdurman Islamic University, Omdurman 14415, Sudan; 6 Department of Pharmaceutics, College of Pharmacy, Prince Sattam Bin Abdulaziz University, Al-Kharj 11942, Saudi Arabia; 7 Department of Pharmaceutics and Industrial Pharmacy, Faculty of Pharmacy, Suez Canal University, Ismailia 41552, Egypt; 8 Department of Pharmaceutics and Pharmaceutical Technology, Faculty of Pharmacy, Al-Azhar University, Cairo 11651, Egypt; 9 Department of Biochemistry, College of Medicine, University of Ha’il, Ha’il 81442, Saudi Arabia; 10 Department of Medical Biochemistry, Faculty of Medicine, Tanta University, Tanta 31511, Egypt; 11 Department of Pharmaceutics and Industrial Pharmacy, Faculty of Pharmacy, Modern University for Technology and Information, Cairo 4410240, Egypt

**Keywords:** anti-bacterial activity, erythromycin, transethosomes, emulgel, cinnamon oil

## Abstract

Erythromycin (EM) is a macrolide antibiotic that is frequently used to treat skin bacterial infections. It has a short half-life (1–1.5 h), instability in stomach pH, and a low oral bioavailability. These foregoing factors limit its oral application; therefore, the development of topical formulations loaded with erythromycin is an essential point to maximize the drug’s concentration at the skin. Accordingly, the current study’s goal was to boost the antimicrobial activity of EM by utilizing the advantages of natural oils such as cinnamon oil. Erythromycin-loaded transethosomes (EM-TE) were generated and optimized using a Box–Behnken design employing, phospholipid concentration (A), surfactant concentration (B), and ethanol content (C) as independent variables. Their effects on entrapment efficiency, EE, (Y_1_) and the total amount of erythromycin that penetrated the skin after 6 h, Q6h (Y_2_), were assessed. The optimized transethosome showed a particle size of 256.2 nm, EE of 67.96 ± 0.59%, and Q6h of 665.96 ± 5.87 (µg/cm^2^) after 6 h. The TEM analysis revealed that, the vesicles are well-known packed structures with a spherical shape. The optimized transethosomes formulation was further transformed into a cinnamon oil-based emulgel system using HPMC as a gelling agent. The generated EM-TE-emulgel was characterized by its physical features, in vitro, ex vivo studies, and antimicrobial activities. The formulation showed sufficient characteristics for effective topical application, and demonstrated a great stability. Additionally, EM-TE-Emulgel had the highest transdermal flux (120.19 μg/cm^2^·h), and showed considerably (*p* < 0.05) greater antimicrobial activity, than EM-TE-gel and placebo TE-Emulgel. The action of EM was subsequently augmented with cinnamon oil, which eventually showed a notable effect against bacterial growth. Finally, these results demonstrate that the transethosomes-loaded cinnamon oil-based emulgel is an alternative way to deliver erythromycin for the treatment of topical bacterial infections.

## 1. Introduction

Erythromycin (EM) is a macrolide antibiotic with a broad spectrum of activity. It is the most commonly used antibiotic for treating skin bacterial infections when administered topically [1]. Erythromycin has a poor water solubility owing to its hydrophobicity, a poor stability in stomach pH, a disagreeable taste, a low oral bioavailability of 35%, and a short half-life (1–1.5 h). These factors limit its oral application and increase the need for development of topical preparations [2]. Although topical drug administration may be advantageous for treating skin conditions, because it lessens systemic adverse effects and increases patient compliance, topical drug administration still has a difficulty in drug delivery since it is challenging to regulate the drug’s permeation through the skin [3]. Therefore, to overcome the aforementioned challenges, transethosomes have been developed, which have demonstrated considerable potential as a delivery system for topically applied active medicinal components.

Nanotechnology is a branch of science that has recently received a lot of attention; it has a variety of applications in the electronics, food, and cosmetic industries, as well as in medicine. The use of nanotechnology in medicine greatly aids the production of novel drug delivery systems for the treatment of illnesses, diagnosis, and therapy [4,5].

Drug delivery systems based on phospholipids have emerged as an effective approach for percutaneous administration of active ingredients due to the biocompatibility and biodegradability of the phospholipid. Nonetheless, the resistive nature of the outermost layer of skin can often prevent medications from passing through the skin. For the successful transfer of drugs through the skin, i.e., getting through the stratum corneum barrier, scientists have explored a variety of approaches. Liposomes were the first developed approach for the dermal delivery of drugs [6]. However, liposomes suffer from a lack of flexibility, which prevented them from penetrating the deeper layers of the skin and forced them to stay in the superficial layer of the skin (epidermis) [7]. Later on, deformable liposomes were developed to address the problems caused by the nature of rigid membranes. The deformable liposomes contain edge activators in addition to phospholipids and water, which increase the flexibility of the liposomes [8] and allow the medication to penetrate the skin more deeply [9]. After that, a new elastic vesicular system, known as ethosomes, was created by Touitou et al. as a follow-up to deformable liposomes [10].

Ethosomes contain ethanol in addition to phospholipids and water, the presence of ethanol distinguishes ethosomes from liposomes. Due to the tendency of ethanol to fluidize the lipid membranes, the flexibility of ethosomes was improved [11] and the penetration of drugs across the epidermis was enhanced. Transethosomes (TE), which contain both edge activators and ethanol, combine the benefits of deformable liposomes with ethosomes [12]. Moolakkadath et al. concluded that transethosomes also showed improved deposition and penetration characteristics [13].

In fact, essential oils and plant extracts have been explored globally as potential sources of novel antimicrobial compounds, therapies for infectious disorders, and food preservation because of their antiviral, antibacterial, and antifungal capabilities. Several studies have been published underscoring the antimicrobial activity of natural products such as lemon grass oil [14], tea tree oil [15], and eucalyptus oil [16].

Cinnamon oil is one of those essential oils whose potential antibacterial properties have been extensively studied [17]. Additionally, it has been asserted that the combination of cinnamon oil and certain antibiotics shows a synergistic action against bacterial species, in comparison to their pure form. El Atki et al. showed that the combination of cinnamon essential oil with certain antibiotics, such as chloramphenicol and ampicillin, has a synergistic antibacterial action against Staphylococcus aureus [18]. Consequently, the purpose of this study was to investigate any potential synergistic antibacterial effects of cinnamon essential oil in combination with certain antibiotics, such as erythromycin, against Staphylococcus aureus, which is the most prevalent bacteria associated with skin infections [19].

This study’s objective was to develop an EM-loaded transethosomes cinnamon oil-based emulgel approach for improving the transdermal delivery of erythromycin and for assessing the effect of the combination between erythromycin and cinnamon oil for efficient antibacterial activity. The erythromycin-loaded transethosomes were developed using Box–Behnken design optimization (BBD). The optimized erythromycin loaded transethosomes were incorporated into a cinnamon oil-based emulgel system using the hydroxypropylmethyl cellulose polymer. Lastly, the obtained preparation was then evaluated for different physical characteristics, in vitro characterization, ex vivo permeation, and antimicrobial efficiency. To the best of our knowledge there has not been much information previously published about EM-loaded transethosomes cinnamon oil-based emulgel formulations.

## 2. Results and Discussion

### 2.1. Exploratory Studies of Erythromycin-Loaded Transethosomes (EM-TE) Preparation

Erythromycin-loaded transethosomes were created utilizing the technique described by Touitou et al., with certain modifications. Preliminary investigations were carried out for choosing a surfactant (edge activator, EA) that generates transethosomes with the highest percentage of entrapment efficiency. It was found that the transethosomal preparation made with Span 80 as the surfactant had a higher entrapment efficiency % (Data not shown), compared to that obtained from Tween 80. These results are relevant to the HLB values of the edge activators. Transethosomes with high entrapment efficiencies are produced using surfactants (EA) with lower HLB values (Span 80 = 4.3), which is due to the higher lipophilicity of the transethosomal vesicles [20]. Our findings were in accordance with Nayak et al., who showed the highest EE% for ketoconazole loaded transethosomes developed from Span 80 [21]. Therefore, Span 80 was chosen as the edge activator in this investigation in order to permit the transethosomes’ membrane flexibility. Subsequently, a Box–Behnken experimental design was employed for erythromycin-loaded transethosomes optimization.

### 2.2. Box–Behnken Design (BBD) for Transethosomes Optimization

The Box–Behnken design produced fifteen experiments for the development of formulations, with three center points. The outcomes of these runs are summarized in Table 1. Entrapment efficiency (Y_1_), and Q6h (Y_2_) are the two dependent variables, and their values ranged from 44.43 ± 1.33% to 82.39 ± 1.53%, and 351.89 ± 11.96 µg/cm^2^ to 819.58 ± 22.05 µg/cm^2^, respectively. The model significance was estimated by ANOVA, it was observed that the quadratic model was the most appropriate model for all responses. Table 2 depicts the R^2^, CV %, and SD values for each of the three dependent responses.

In addition, Table 2 demonstrates the data linearity, by showing a linear correlation between the adjusted and predicted R^2^ results for the all-dependent responses. Furthermore, the model’s validity was confirmed by the lack of fit in the dependent responses, which revealed insignificant values (F-value) of 2.20 and 0.4580, and *p*-values of 0.3272 and 0.7401, for Y_1_ and Y_2_ (*p* > 0.05), respectively. Additionally, the low coefficient variation (% CV) and high adjusted R^2^ (adj) for Y_1_ and Y_2_ further showed that we had effectively created a model with high accuracy, reproducibility, and reliability for optimizing the transethosomal formulation.

#### 2.2.1. Effect of Independent Factors on the EE% (Y_1_)

As shown in Table 1, the entrapment efficiencies ranged from a minimum value of 44.43 ± 1.33% (F9) to a maximum value of 82.39 ± 1.53% (F13). The following polynomial Equation (1) revealed that the phospholipid concentration (A) had a synergetic influence on the drug EE%, whereas the surfactant concentration (B) and ethanol concentration (C) had a negative impact.

(1) $$Y_{1}=62.24+5.68A\;-7.38B\;-9.75\;C\;+2.4\mathrm{AB}\;-1.75\mathrm{AC}\;+2.37\;\mathrm{BC}\;-1.71{\;A}^{2}+2.66{\;B}^{2}-1.72{\;C}^{2}\;$$

The increase in the encapsulation efficiency with an increase in the phospholipid concentration could be related to the lipophilicity of the drug, since the lipophilic medication will be directed to be encapsulated in the lipoid phase of the vesicles [22]. As shown in Table 1, the formulation (F1) which contained 4% of phospholipid demonstrated an entrapment efficiency of 73.11 ± 0.74%, compared to the formulation (F7), containing 2% of phospholipid, exhibiting an entrapment efficiency of 67.46 ± 0.48%. Similar findings were shown for formulation (F4), with 4% phospholipid (entrapment efficiency 52.53 ± 2.33%), and formulation (F9) with 2% phospholipid (entrapment efficiency 44.43 ± 1.33%). Also, formulation (F2) (entrapment efficiency 64.41 ± 0.89%) produced a higher entrapment efficiency compared to formulation (F6) (entrapment efficiency 47.79 ± 1.19%). These findings are consistent with those obtained by Abdallah et al. who indicated that, the increase in lecithin concentration led to an increase in the EE% of Brucine-loaded ethosomes. [23].

**Figure 1:**
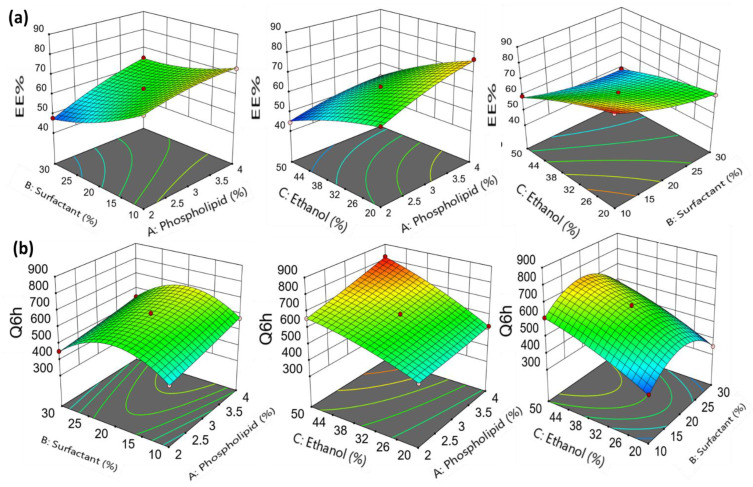
3D-response surface plot showing the effect of independent variables on (**a**) Entrapment efficiency (Y_1_) and (**b**) Cumulative amount of drug permeated after 6 h (Q6h) (Y_2_).

According to Figure 1a, the percent of entrapment efficiency reduced as the surfactant concentration increased. The entrapment efficiency of formulation (F3) (surfactant 10%) was 59.31 ± 0.92%, while for formulation (F8) (surfactant 30%) it was 48.73 ± 1.45%. In addition, both formulations (F1, 10% surfactant, and F2, 30% surfactant) produced similar findings (entrapment efficiency 73.11 ± 0.74% vs. 64.41 ± 0.89%, respectively). Similar findings were observed between formulation (F7) (EE of 67.46 ± 0.48%) and formulation (F6) (EE of 47.79 ± 1.19%). An increase in the surfactant concentration may develop pores in the vesicles bilayers, making them leaky to the drug that is encapsulated [24]. Additionally, a higher surfactant concentration triggered the development of mixed micelles coexisting with the formulated transethosomal vesicles [20].

As illustrated in Figure 1a, ethanol had a negative impact on the EE% of erythromycin in transethosomes. The entrapment efficiency for formulation (F5), with 20% ethanol, was 61.61 ± 0.42%, but for formulation (F9), with 50% ethanol, it was 44.43 ± 1.33%. The entrapment efficiency was shown to be 82.39 ± 1.53% for formulation (F13), with 20% ethanol, and 59.31 ± 0.92% for formulation (F3), with 50% ethanol. Similar findings were observed between formulation (F11) (EE of 76.69 ± 0.47%) and formulation (F4) (EE of 52.53 ± 2.33%) (Table 1). This reduction in the entrapment efficiency with ethanol concentration increase could be related to the higher vesicle fluidity, which increased the leakiness of the vesicles and led to drug leakage [25]. These results are consistent with those obtained by Moolakkadath et al., who demonstrated that ethanol had a negative impact on the encapsulation of fisetin-loaded transethosomes [13].

**Figure 2:**
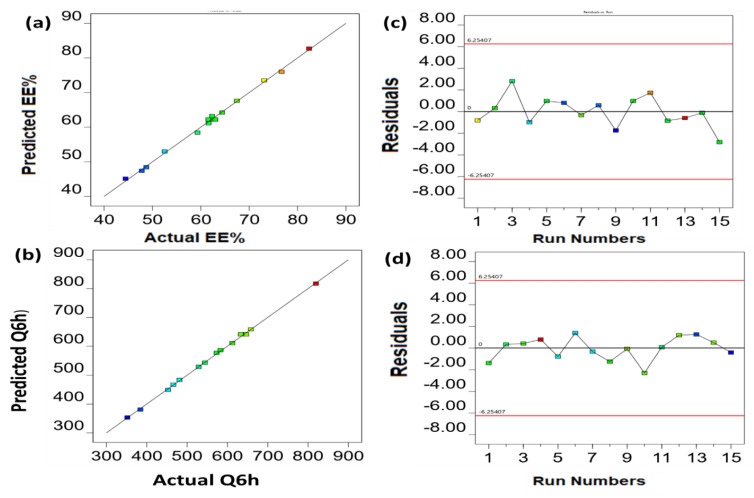
Linear correlation plots (**a**,**b**) between predicted and actual values and (**c**,**d**) the corresponding residual plots for different responses.

Figure 1a shows the three-dimensional response surface graph, which determines the impact of the independent variables on the entrapment efficiency. In addition, Figure 2a,c demonstrates that the data are linear, since the predicted R^2^ value (0.9632) correlates with the adjusted R^2^ value (0.9920).

#### 2.2.2. Effect of Independent Variables on the Cumulative Amount of Drug Permeated after 6 h (Q6h) (Y_2_)

As shown in Table 1, the cumulative amount of drug permeated after 6 h (Q6h) ranged from a minimum value of 351.89 ± 11.96 (F15) to a maximum value of 819.58 ± 22.05 (F4).

(2) $$Y_{2}=641.24+50.92\;A-12.98\;B\;+115.84\;C-4.00\;\mathrm{AB}+28.44\;\mathrm{AC}-0.82\;\mathrm{BC}\;+3.46{\;A}^{2}-135.64{\;B}^{2}-22.72{\;C}^{2}\;$$

Equation (2) shows that, the concentration of phospholipids had a positive impact on the cumulative amount of drug permeated after 6 h (Q6h). It was shown that formulation (F1) (phospholipid, 4%) had a greater Q6h, of 573.21 ± 36.38 µg/cm^2^, than formulation (F7) (phospholipid, 2%), which had a Q6h of 466.04 ± 22.72 µg/cm^2^. Additionally, similar findings were shown across formulations (F4) and (F9), and formulation (F2) (phospholipid, 4%) and formulation (F6) (phospholipid, 2%) (Table 1). The increase in the cumulative amount of drug penetrated with increasing vesicle phospholipid concentration could be due to the ability of lipids to enhance the vesicles’ partition through the skin lipid barrier.

Similarly, ethanol also demonstrated a positive impact on the erythromycin permeation via skin. It was observed that, the cumulative amount of drug permeated after 6 h for formulation (F5), with 20% ethanol, was 481.24 ± 22.35 µg/cm^2^, but for formulation (F9), with 50% ethanol, it was 658.21 ± 18.04 µg/cm^2^. Likewise, Q6h was shown to be 384.33 ± 10.95 µg/cm^2^ for formulation (F13), with 20% ethanol, and 612.22 ± 26.65 µg/cm^2^ for formulation (F3), with 50% ethanol (Table 1).

The increase in ethanol concentration resulted in an increase in the drug permeation via the skin, which could be related to the ethanol’s penetration enhancing features. In addition, ethanol has the ability to lower the lipid’s transition temperature, imparts flexibility to the vesicles, and permits the vesicles to penetrate through the skin [10]. Additionally, it is possible that ethanol promotes fluidization of the stratum corneum, which could increase the penetration of flexible vesicles through it, by reacting with the polar head groups of the lipid molecules in the stratum corneum [26].

The cumulative amount of drug permeated after 6 h increased when increasing the surfactant concentration from 10 to 20%, as shown in Figure 1b. Following this, the drug permeation for formulations containing 20 to 30% of Span 80 gradually decreased (Figure 1b). First, the higher surfactant concentration could have altered the bilayer’s packing properties, which could result in the production of less organized and more leaky vesicles’ membrane, hence the drug permeation was increased [20]. Following that, the permeation was reduced with increasing surfactant concentrations, which could be related to the formation of micelles at higher Span 80 concentrations [13]. Figure 1b shows the three-dimensional response surface graph, which determines the impact of the independent variables on the cumulative amount of drug permeated after 6 h. In addition, the linearity of the data was supported by the linear correlation plot shown in Figure 2b,d, where the predicted R^2^ value was 0.9933 and the adjusted one was 0.9976.

#### 2.2.3. Selection of the Optimized Formulation of Erythromycin Loaded Transethosomes (EM-TE)

**Figure 3:**
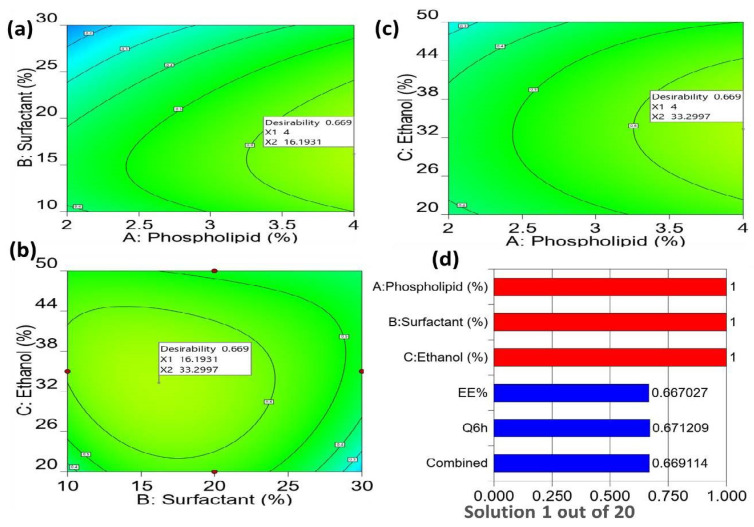
2D contour plot for desirability function displaying the effect of independent factors on the overall responses (**a**–**c**); and desirability bar graph displaying the overall desirability of each response and combined optimization (**d**).

Utilizing the Design Expert^®^ software, the optimum preparation, with the requisite characteristics, was specified after the development of the BBD using the point prediction method. By adjusting the criteria in favor of maximum values of EE% (Y_1_) and Q6h (µg/cm^2^) (Y_2_), the optimal formula was chosen from the fifteen experiments (Figure 3). It was revealed that the transethosomal formulation which contains 4% of phospholipid, 16.20% of Span 80, and 33.3% of ethanol, satisfied the criteria for an ideal formulation. The optimal formulation had an entrapment efficiency of 67.96 ± 0.59% and a cumulative amount of drug permeated of 649.99 ± 13.62 (µg/cm^2^) after 6 h. These values are close to those predicted by the BBD. The predicted value for the entrapment efficiency was 69.74 ± 0.95% and for the cumulative amount of drug permeated was 665.96 ± 5.87 (µg/cm^2^) after 6 h. These results supported the generated response surface methodology model’s validity.

### 2.3. Characterization of the Optimized Erythromycin Loaded Transethosomes (EM-TE)

**Figure 4:**
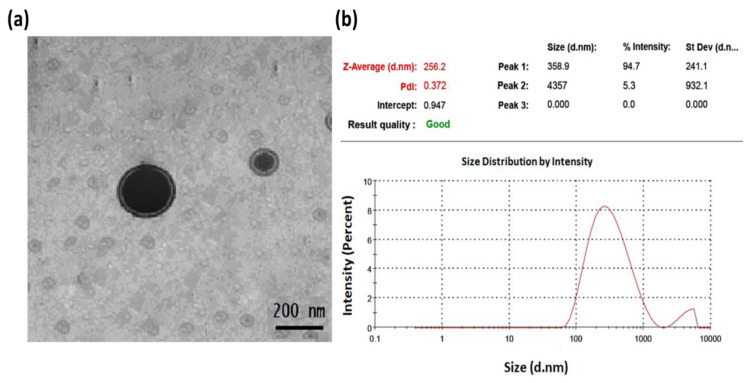
Transmission electron micrograph of optimized erythromycin transethosomes formulation (**a**) and vesicle size distribution curve (**b**).

The TEM examination of the optimized transethosomes showed that the erythromycin-loaded transethosomes were well-recognized packed structures with a spherical shape and consistent size distribution (Figure 4a). The size distribution curve of the transethosomes formulation with optimum erythromycin loading is shown in Figure 4b. The average particle size was 256.2 nm with a PDI of 0.372. The vesicle size distribution confirms that they are distributed normally.

### 2.4. In Vitro Characterization of Erythromycin-Loaded Transethosomal Emulgel

#### 2.4.1. Homogeneity Organoleptic Test

Erythromycin-loaded transethosomal emulgel was evaluated for a variety of variables including consistency, homogeneity, clarity, and pH. The prepared formulation was physically and visually examined for color changes, consistency, phase separations, and liquefaction as part of the homogeneity organoleptic test. The developed formulation had a smooth homogenous consistency and an off-white color. The pH value of the generated formulation was 6.1 ± 0.4, which was considered safe and avoided causing any irritation on skin application.

#### 2.4.2. Determination of Extrudability and Spreadability Determination

Extrudability and spreadability are regarded as the most important features for patient compliance and the homogenous distribution of topical formulations. The produced transethosomal emulgel had an extrudability and spreadability of 169.87 ± 4.60 gm/cm^2^ and 5.80 ± 0.46 cm, respectively. These findings suggest that the produced formulation has an excellent spreadability rate across the skin’s surface and would be easily extruded by applying pressure with the thumb.

#### 2.4.3. Drug Content Assessment

One of the most important conditions for any kind of dosage form is identifying the content of the drug. According to Garala et al., the precise amount of bioactive agent in any formulation should not deviate too much from the amount specified on the label [27]. The erythromycin-loaded transethosomal emulgel has a 96.52 ± 3.04% drug content percentage. This result showed that the drug content was within the permitted range (100 ± 10%). This demonstrated that the drug was distributed uniformly throughout the transethosomal emulgel.

#### 2.4.4. Viscosity

Viscosity is a crucial physical characteristic of topical preparations and has a significant impact on drug release. The viscosity additionally influences the stability, spreadability, drug release, and simplicity of application of semisolid dosage forms [28]. The viscosity of the erythromycin-loaded transethosomal emulgel was (5798.67 ± 295.2 cp), which is acceptable and within the normal range.

### 2.5. Studies of the In Vitro Release of Erythromycin from Cinnamon Oil Based Transethosomal Emulgel

**Figure 5:**
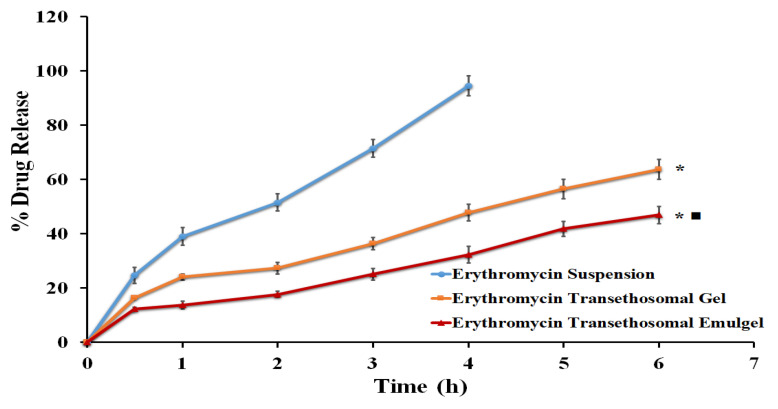
In vitro release study of erythromycin from different formulations compared to erythromycin suspension. Results are represented as the mean ± SD of three runs. * *p* < 0.05 compared to erythromycin suspension; and ■ *p* < 0.05 compared to erythromycin transethosomal gel.

Figure 5 shows the in vitro erythromycin release profiles from the formulated cinnamon oil-based transethosomal emulgel, and from transethosomal gel at pH 7.4 using phosphate buffer. The percentage of erythromycin released after 6 h was 46.92 ± 3.15%, 63.69 ± 3.68% for the transethosomal emulgel and transethosomal gel, respectively, but the free erythromycin showed a drug release of 94.54 ± 3.64% after 4 h. It is obvious that both formulations produced much less erythromycin than the free drug (*p* < 0.05). The use of a gelling ingredient, which increases the viscosity or thickness of the formulations and hence inhibits the release of the drug from the prepared formulations, could explain the noticeably reduced erythromycin release from gel and emulgel formulations when compared to the free drug. Additionally, the percentage release of the drug from transethosomal gel was much higher than the percentage drug release from transethosomal emulgel (*p* < 0.05). The gel formulation has a high water content, which makes the drug diffuse into the release medium easily [29].

By contrast, in comparison to the transethosomal gel formulation, the much lesser release of erythromycin from the transethosomal emulgel was attributed to the formulation’s higher viscosity, which could prevent the effective diffusion of the encapsulated drug. Additionally, the reduced water content and the incorporation of cinnamon could explain the lower drug release [30].

### 2.6. Skin Permeation Study

**Figure 6:**
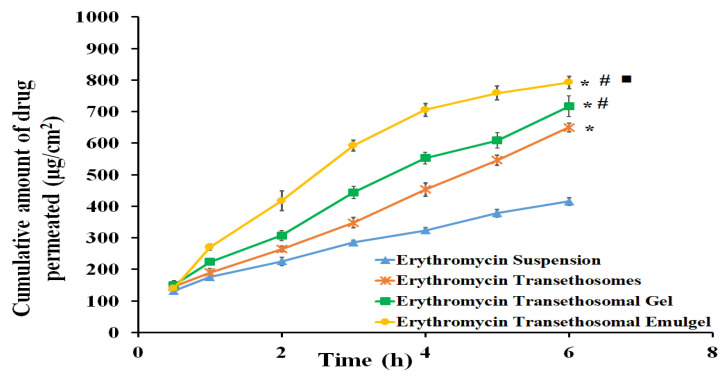
Permeation study of erythromycin from different formulations compared to erythromycin suspension (control). Results are represented as mean ± SD (n = 3). * compared to Erythromycin suspension (*p* < 0.05); # compared to erythromycin transethosomes (*p* < 0.05); and ■ compared to erythromycin transethosomal gel (*p* < 0.05).

Figure 6 indicates the percutaneous penetration of erythromycin through rabbit skin from transethosomal gel and transethosomal emulgel formulations in comparison to the permeation from an erythromycin suspension. The drug was observed to penetrate the rabbit skin in the following order: erythromycin-loaded transethosomal emulgel > erythromycin-loaded transethosomal gel > transethosomes > erythromycin suspension. Additionally, the cumulative amount of erythromycin permeated from the transethosomal emulgel through the rabbit skin (792.48 ± 18.82 μg/cm^2^) was significantly higher than that from the transethosomal gel (716.93 ± 33.26 μg/cm^2^), transethosomes (649.99 ± 13.62 μg/cm^2^), or erythromycin suspension (415.13 ± 11.29 μg/cm^2^), as shown in Figure 6. According to our findings, all of the formulations under consideration had SSTF values that were much higher than that of the erythromycin suspension (50.77 μg/cm^2^·h). However, the erythromycin-loaded transethosomal gel increased the drug permeability by two times, with an SSTF of 102.14 μg/cm^2^·h, which could be related to the gel formulation’s colloidal characteristics. Curiously, the erythromycin-loaded emulgel, which had the highest SSTF value (120.19 μg/cm^2^·h), was able to considerably increase the skin permeation characteristics of erythromycin (*p* < 0.05) when compared to other formulations. The dual influences of surfactants in the transethosomal and emulsion formulations could also be the cause of the higher drug permeability through the skin of the transethosomal emulgel. Our results are in accordance with Surini et al., who proved that transfersomes improve drug permeation through the skin [31].

Moreover, the emulgel contained cinnamon oil acting as a penetration enhancer [32], which might increase the drug permeation from the emulgel and would result in a 2.4-fold increase in permeability. On the other hand, the results showed that, the drug permeability from transethosomes (SSTF, 91.62 μg/cm^2^·h) was less when compared to the transethosomal gel and emulgel, this may be due to the interaction with skin through the effect of transethosomes’ composition only [33].

### 2.7. Studies of Stability

**Figure 7:**
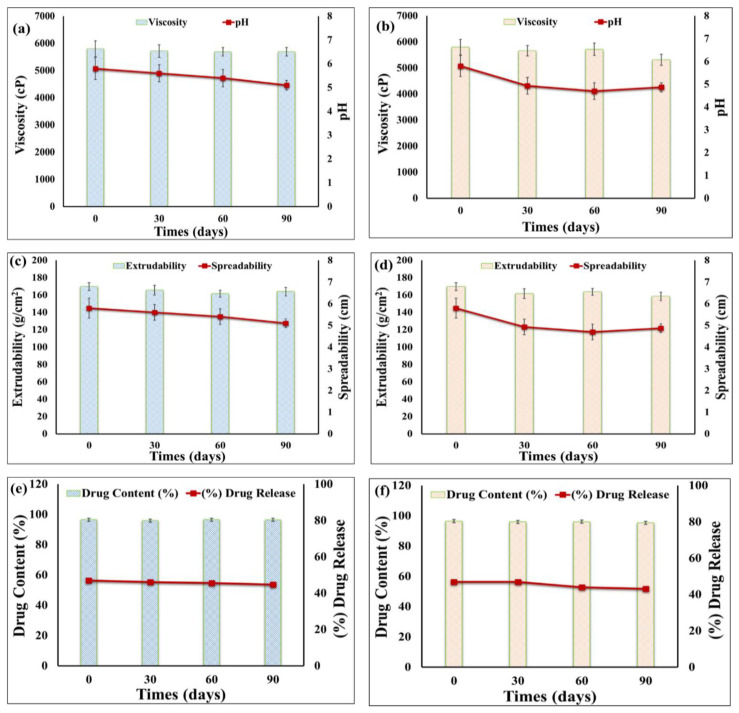
Outline of stability study for erythromycin-loaded cinnamon oil-based transethosomal emulgel in comparison to freshly prepared formulation at 25 °C (**a**,**c**,**e**) and 4 °C (**b**,**d**,**f**). The effects were determined on pH and viscosity (**a**,**b**), extrudability and spreadability (**c**,**d**), percentage of drug content and percentage of drug release (**e**,**f**).

The physical properties and the percentage release of erythromycin from the prepared transethosomal emulgel were recorded during a three-month period at 4 °C and 25 °C, and the results are shown in Figure 7a–f. The findings show that there were no significant changes in the color, homogeneity, content uniformity, pH, viscosity, extrudability, and spreading of the formulation after being stored at both temperatures. Additionally, as compared to the corresponding fresh preparations, there was no appreciable differences in the percentage of drug content and percentage release of erythromycin from the formulation being stored at 4 or 25 °C (*p* < 0.05). Therefore, the findings confirmed that the erythromycin-loaded cinnamon oil-based transethosomal emulgel was incredibly stable over the allotted time.

### 2.8. Antimicrobial Activity

**Figure 8:**
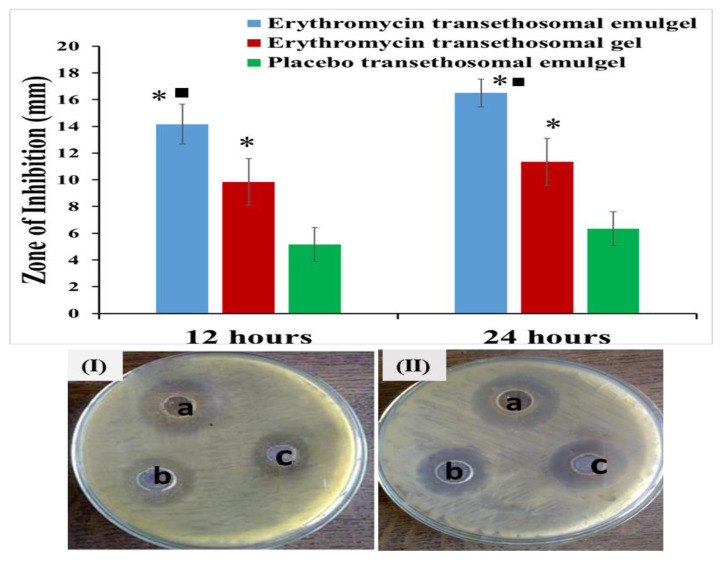
Comparative antimicrobial results of (a) Erythromycin-loaded transethosomal emulgel (EM-TE-Emulgel), (b) Erythromycin-loaded transethosomal gel (EM-TE-Gel) and (c) placebo transethosomal emulgel against *S. aureus* after 12 h (**I**) and 24 h (**II**). Results are represented as mean ± SD (n = 3). * compared to placebo transethosomal emulgel (*p* < 0.05); and ■ compared to Erythromycin transethosomal gel (*p* < 0.05).

The findings of the antibacterial evaluation study utilizing the cup plate method on *S. aureus* (Gram-positive bacteria) are shown in Figure 8. The erythromycin-loaded transethosomal emulgel (EM-TE-Emulgel) demonstrated zones of inhibition of 14.5 ± 1.04 mm (after 12 h) and 16.5 ± 1.5 mm (after 24 h) against *S. aureus*. The erythromycin-loaded transethosomal gel (EM-TE- Gel) demonstrated zones of inhibition of 9.83 ± 1.70 (12 h) and 11.33 ± 1.75 mm (24 h) against *S. aureus*. Additionally, the placebo transethosomal emulgel (TE-Emulgel) revealed that the zones of inhibition were 6.33 ± 1.26 mm and 5.17 ± 1.25 mm in 12 h and 24 h, respectively, against *S. aureus*. As previously mentioned, erythromycin has the ability to prevent protein synthesis by blocking the production of peptide chains through binding with ribosomal RNA molecule of the bacterial ribosome [34].

The findings show that the activity of the EM-TE-Emulgel was significantly (*p* < 0.05) higher than that of the EM-TE-Gel and the placebo TE-Emulgel. It is noteworthy that the placebo TE-Emulgel formulation, including cinnamon oil, showed a significant suppression for bacterial growth, which was certainly attributable to the cinnamon oil antibacterial action. Cinnamon oil has been shown to have antimicrobial action against the tested bacteria, and these findings are mostly consistent with other studies [17]. The observed greater antimicrobial efficiency of cinnamon essential oil could be attributed to the effect of cinnamaldehyde, which is recognized as the main component in cinnamon oil [18]. There have been reports that cinnamon essential oils have the ability to inhibit bacterial growth through cell membrane damage, lipid profile changes, inhibition of ATPases, inhibition of cell division, inhibition of motility, and inhibition of the formation of biofilms [35].

The antibacterial synergism between EM and cinnamon oil could be responsible for the enhanced antibacterial activity of EM-TE-Emulgels. There were some investigations which identified the antimicrobial efficiency of cinnamon essential oil in combination with other antibiotics; Mahadlek et al. proved that cinnamon essential oil exhibited synergistic effects against *S. aureus* when combined with metronidazole, ciprofloxacin hydrochloride, or doxycycline hyclate [36]. Our current study supported these findings, demonstrating the effectiveness of cinnamon oil in combination with erythromycin.

## 3. Conclusions

Erythromycin was encapsulated successfully in transethosomes, which is thought to be a promising technique of drug delivery. Several transethosomal formulas were developed using the Box–Behnken design approach. The optimum formula, which contained 4% phospholipid, 16.20% Span 80, and 33.3% ethanol, was chosen based on the values of certain studied parameters. The optimized transethosomal formulation was introduced into an emulgel for easier application. The transethosomal emulgel formulation had acceptable physical characteristics that were suited for topical use in addition to an effective percutaneous absorption through the skin. The transethosomal emulgel with cinnamon oil notably displayed a significant antibacterial activity that enhances erythromycin’s ability to prevent the growth of bacteria. In summary, the investigation noted that combining erythromycin transethosomes with an emulgel formulated with cinnamon oil, synergistically improved the antibacterial action of erythromycin.

## 4. Materials and Methods

### 4.1. Materials

Erythromycin (EM), Span 80, Tween 80, propylene glycol, ethyl alcohol, Phospholipone H 100 (Pl), and hydroxypropylmethyl cellulose (HPMC) were procured from Sigma-Aldrich (St. Louis, MO, USA). Cinnamon oil was procured from NOW^®^ Essential oils (NOW Foods, Bloomingdale, IL, USA). All other chemicals were of analytical grade.

### 4.2. The Experimental Design Study

Design expert software (Version 12, Stat-Ease Inc., Minneapolis, MN, USA) was implemented to exhibit the response surface model by accomplishing various combinations of values. A three-factor, three-level (3^3^) Box–Behnken design (BBD) was used in order to manufacture several transethosomal (TE) formulations. The effects of three formulation factors, namely phospholipid concentration (A), surfactant concentration (B), and ethanol concentration (C), on two formulation’s characteristics, namely, entrapment efficiency EE% (Y_1_) and cumulative amount of drug permeated after 6 h, Q6h (Y_2_) of the developed transethosomes were evaluated. The independent factors were assessed at various levels (−1, 0, 1). Table 3 provides a summary of the experimental design pattern for the Box–Behnken design. The determination of the optimal formula and the desired characteristics were dependent on the development of 15 runs with three central points. The significance of the model was evaluated using ANOVA, the analysis of variance test, which also served for validation of the statistical analysis’ findings. In order to correlate the results from the statistical analysis, three-dimensional surface plots were developed.

### 4.3. Development of Erythromycin Transethosomal Formulations (EM-TE)

The transethosomal dispersions containing erythromycin (EM) were created by modifying the Touitou et al. methodology, which is the most straightforward and widely used method for generating transethosomal systems [37]. The aqueous and organic phases were made independently. The specified amount of phospholipone H 100 (Pl), surfactant (Span 80, edge activator), and EM were dissolved in ethanol (organic phase) at room temperature while being vigorously stirred at 1200 rpm utilizing a magnetic stirrer. An aqueous phase, made of distilled water and one-milliliter propylene glycol (PG), was added drop-by-drop to the organic phase using a syringe. The mixture was then stirred for thirty minutes at 700 rpm to generate the desired transethosomal dispersion (TE). The produced dispersion was homogenized three times for five minutes using a homogenizer (Ika-eurostar 20 high speed digital, Germany). The prepared transethosomal formulations were kept in the fridge and were tested for particle size, entrapment efficiency %, and cumulative amount of drug permeated after 6 h (Q6h). Table 1 lists the constituents of the obtained transethosomal formulations based on the various evaluated characteristics.

### 4.4. Characterization of the Generated Erythromycin Transethosomal Formulations (EM-TE)

#### 4.4.1. Entrapment Efficiency (EE%)

The centrifugation technique was utilized to determine the % of EM entrapped within transethosomal formulations. The obtained formulation was centrifuged for one hour at 6000 rpm using a centrifuge (TGL-16 Tabletop High Speed Refrigerated Centrifuge, China) [23]. The supernatant was then withdrawn and spectrophotometrically quantified for the presence of the free drug utilizing a spectrophotometer (U.V. Spectrophotometer, Uviline 9100, SCHOTT-EU) at a maximum wavelength of 280 nm [38]. The EE% was computed using the following formula:

$$\mathrm{Drug}\;\mathrm{EE}\;\left(\%\right)=\left[\left(\mathrm{Total}\;\mathrm{EM}-\;\mathrm{Free}\;\mathrm{EM}\right)/\mathrm{Total}\;\mathrm{EM}\right]\times 100$$

#### 4.4.2. Ex Vivo Skin Permeation Study of EM from the Developed Transethosomal Formulations

First, an electric clipper was used to remove the hair from the abdomen region of white albino male rabbits. The skin of the animal was then removed after it had been sacrificed. The separated skin was maintained in the refrigerator until further investigation, where it was hydrated with PBS (pH 7.4) containing 0.02% sodium azide overnight at 4 °C [39].

Modified diffusion cells that were set up in our lab were used to conduct a permeation investigation [23]. The skin membranes were connected to the receptor medium, which included one hundred milliliters of hydration media (PBS, pH 7.4), and adjusted at 37 ± 0.5 °C. The donor section was filled with erythromycin transethosomal formulations (EM-TE) and was linked to glass tubes with a 6.15 cm^2^ permeation area [40]. Samples of about one milliliter were taken at specific periods for 6 h. The same volume of fresh blank phosphate buffer was introduced in order to keep the volume of the receptor media uniform. Using a spectrophotometer, the samples were assessed at a maximum wavelength (λ_max_) of 280 nm.

### 4.5. Characterization of the Optimized Erythromycin Transethosomal Formulations (EM-TE)

The optimized formulation was selected based on the results obtained from the experimental design utilizing Design Expert, and the optimized formulation was further examined. The morphology of the optimized formulation was studied utilizing the transmission electron microscope TEM (JEOL, JEM, Tokyo, Japan). A drop of freshly made transethosomal dispersion was placed on a copper grid that had been covered with carbon. The sample was colored with 2% ammonium molybdate, and allowed to dry for fifteen minutes at room temperature, then viewed under appropriate magnifications using TEM. Additionally, the vesicle size and PDI of the optimized erythromycin transethosomes (EM-TE) was examined using a Zetasizer device (Malvern Instruments Ltd., Worcestershire, UK) [20].

### 4.6. Preparation of Transethosomal Cinnamon Oil-Based Emulgel Loaded with Erythromycin

The optimized transethosomes (EM-TE) were incorporated into an emulgel loaded with cinnamon oil. First, the aqueous gel polymeric base was manufactured, using (4% *w/w*) HPMC as a gelling agent. The hydrogel base was generated using a magnetic stirrer (Magnetic Stirrer–AREC, VELP Scientifica, Milano, Italy) by gently dispersing a pre-determined quantity of HPMC over distilled water (ten milliliters) and properly mixing until a homogenous hydrogel base was obtained [41]. On the other hand, one gram of cinnamon oil was mixed with one gram of Tween 80 for five minutes using a vortex mixer (Fisher Scientific, USA). The aqueous phase was then gently mixed with the oily phase while being vortexed for ten minutes until obtaining a white emulsion. The obtained emulsion was gently introduced to the prepared hydrogel base and properly stirred for five minutes to develop a uniform cinnamon oil-based emulgel [42]. For generating transethosomal emulgel containing erythromycin (EM-TE-Emulgel), the pre-formulated transethosomal preparation was blended with the generated cinnamon oil-based emulgel utilizing a vortex mixer (Fisher Scientific, USA) until achieving the required formulation [43]. A transethosomal gel (TE-Gel) containing erythromycin (EM) was developed to confirm the impact of the emulsion and cinnamon oil in our investigation. In which, the aqueous gel base was then mixed directly with the specified amount of transethosomal formulation to get the erythromycin transethosomal gel (EM-TE-Gel).

### 4.7. In Vitro Characterization of Erythromycin-Loaded Transethosomal Emulgel

#### 4.7.1. Organoleptic Evaluation and pH Determination

The appearance and homogeneity of the generated formulations were examined visually and checked for the presence of any grittiness and agglomerates [29]. Moreover, by using a pH meter (PCT-407 Portable pH Meter, Taipei City, Taiwan), the pH value of the developed formulation was assessed at room temperature [44].

#### 4.7.2. Determination of the Extrudability and Spreadability

The assessment of spreadability and extrudability is a crucial factor in determining the uniform distribution and spreading of the prepared formulation [45]. The spreadability defines the area where the generated formulations may readily spread after skin application. Among two glass slides (20 cm × 20 cm), one gram of the tested preparations was placed, and a standard load (500 g) was introduced on the upper slide for five minutes [46]. The spreading area’s diameter was measured to determine the spreadability parameter [47]. The extrudability is the weight in grams required to extrude at least a half-centimeter ribbon of the formulation from a collapsible tube in ten seconds. The EM-loaded transethosomal emulgel (10 g) was packed in a collapsible tube, and the crimped end of the tube was pressed to cause the gel to extrude upon removal of the cap [48]. The extrudability (g/cm^2^) was determined by employing the equation shown below:

$$\mathrm{Extrudability}=\frac{\mathrm{Applied}\;\mathrm{weight}\;\mathrm{to}\;\mathrm{extrude}\;\mathrm{gel}\;\mathrm{from}\;\mathrm{tube}\;\left(\mathrm{Gram}\right)\;}{\mathrm{Area}\;\left({\mathrm{cm}}^{2}\right)}$$

#### 4.7.3. Drug Content Assessment

A precise volume of ethanol was used to dilute half a gram of the manufactured emulgel formulation (equal to 10 milligrams of erythromycin) to ten milliliters. The mixture was stirred for one hour following filtration through Whatman filter paper. The content of the drug was spectrophotometrically examined at a maximum wavelength of 280 nm against a blank sample made up of the identical components but without the drug. The drug content was calculated as a percentage [49].

#### 4.7.4. Viscosity Measurement

A Brookfield viscometer (Brookfield viscometer, Model DV-II, spindle number 02, Middleboro, MA, USA) was used to assess the viscosity of the produced transethosomal emulgel at 25 ± 1 °C. A certain amount of the prepared formulation was introduced in a beaker and permitted to settle at room temperature for thirty minutes. The spindle was lowered into the formulation without touching the beaker bottom, and revolved at 20 rpm. The viscosity measurements were taken three times, and the average of the three measurements was reported [50,51]. The viscosity in centipoises (cP) was calculated.

### 4.8. In Vitro Release of EM from Transethosomal Emulgel

Using the technique previously described by Ibrahim et al. [29], the in vitro release profile of erythromycin from different preparations was assessed utilizing USP dissolution apparatus II with certain modifications. In brief, glass tubes with a cellophane membrane (MW cut-off 12,000–14,000 Da) attached to one end were filled with half of a gram of formulations containing 10 mg of drug. The glass tubes were dipped in 100 mL of phosphate buffer (pH 7.4) and rotated at 50 rpm at 37 ± 1.0 °C. Throughout the experiment, samples were periodically withdrawn and replaced with an equivalent amount of fresh dissolution media to maintain the sink’s condition. The obtained samples were assayed using spectrophotometer at a maximum wavelength (λ_max_) of 280 nm.

### 4.9. Skin Permeation Study from Transethosomal Emulgel

A modified diffusion cell was used to conduct the skin permeation investigation. The receptor medium was comprised of 100 mL of phosphate buffer (pH 7.4) and 0.02% sodium azide and adjusted to be at 37 ± 1.0 °C. The skin membrane was attached to the receptor media. The amount of erythromycin that permeated from the prepared formulation was measured using the same procedure as previously mentioned in Section 4.4.2. The ex vivo permeation characteristics of erythromycin, including steady state transdermal flux (μg/cm^2^·h) and enhancement ratio, were calculated.

$$\mathrm{Steady}\;\mathrm{state}\;\mathrm{transdermal}\;\mathrm{flux}\;\left(\mathrm{SSTF}\right)=\frac{\mathrm{Amount}\;\mathrm{of}\;\mathrm{drug}\;\mathrm{penetrated}\;\mathrm{the}\;\mathrm{skin}\;}{\mathrm{area}*\;\mathrm{time}}$$

$$\mathrm{Enhancement}\;\mathrm{ratio}\;\left(\mathrm{ER}\right)=\frac{\mathrm{SSTF}\;\mathrm{from}\;\mathrm{experiment}\;}{\mathrm{SSTF}\;\mathrm{from}\;\mathrm{control}}$$

### 4.10. Stability Study of the Generated Transethosomal Emulgel

The viscosity, pH, extrudability, spreadability, drug content, and percentage of in vitro drug release after 6 h were all measured to determine the stability of the produced erythromycin-loaded cinnamon oil-based transethosomal emulgel. The investigated emulgel was stored for 90 days at two distinct temperatures, 25 ± 0.5 °C and 4 ± 0.5 °C. These variables were examined routinely at 0, 30, 60, and 90 days [29].

### 4.11. Antimicrobial Activity

The antibacterial activity of the prepared erythromycin-loaded transethosomal emulgel (EM-TE-Emulgel) was evaluated, using the cup plate technique, against Staphylococcus aureus (*S. aureus*). The cup plate technique was used based on the experiment of El Atki et al. with certain modifications [18]. The plates were first sterilized for 60 min in a hot air oven at 160 °C. Each plate was then filled with 10 mL of sterile nutrient agar media (sterilized for 15 min at 121 °C in an autoclave). The plates were permitted to harden under aseptic conditions, then the micro-organism was inoculated into the plates [38]. Three cups, each 3 mm in diameter, were created in the plate using a cork borer, and the samples were added to evaluate their efficacy. The plates were then incubated at 25 °C for 24 h and the zone of inhibition was assessed for each preparation using a graduated scale, and the obtained data were compared.

### 4.12. Statistics

The acquired data were reported as means ± SD (n = 3). One-way ANOVA was used in the statistical analysis using GraphPad Prism version 5. If *p* < 0.05, the difference was regarded as statistically significant.

## Figures and Tables

**Table 1:** The values of independent factors utilized for optimization of transethosomal preparations and the determined outcomes of dependent variables.

Formulation	Independent Variables			Dependent Variables	
	A (%)	B (%)	C (%)	Y_1_ (%)	Y_2_ (µg/cm^2^)
F1	4	10	35	73.11 ± 0.74	573.21 ± 36.38
F2	4	30	35	64.41 ± 0.89	544.06 ± 28.53
F3	3	10	50	59.31 ± 0.92	612.22 ± 26.65
F4	4	20	50	52.53 ± 2.33	819.58 ± 22.05
F5	2	20	20	61.61 ± 0.42	481.24 ± 22.36
F6	2	30	35	47.79 ± 1.19	452.90 ± 20.48
F7	2	10	35	67.46 ± 0.48	466.04 ± 22.72
F8	3	30	50	48.73 ± 1.45	583.07 ± 20.25
F9	2	20	50	44.43 ± 1.33	658.21 ± 18.04
F10	3	20	35	63.01 ± 0.83	633.16 ± 26.68
F11	4	20	20	76.69 ± 0.47	528.87 ± 18.86
F12	3	20	35	61.57 ± 0.92	646.71 ± 27.02
F13	3	10	20	82.39 ± 1.53	384.33 ± 10.95
F14	3	20	35	62.15 ± 0.45	643.84 ± 17.78
F15	3	30	20	62.33 ± 0.16	351.89 ± 11.96

A—Phospholipid concentration (%); B—surfactant concentration (%); C—ethanol concentration (%); Y_1_, Entrapment efficiency (%); Y_2_, Cumulative amount of drug permeated after 6 h (µg/cm^2^).

**Table 2:** Regression analysis results using ANOVA for independent responses Y_1_ and Y_2_ using quadratic model fitting.

Source	Y_1_		Y_2_	
	F-Value	*p*-Value	F-Value	*p*-Value
Model	192.96	<0.0001 *	652.54	<0.0001 *
A—Phospholipid (%)	285.50	<0.0001 *	602.81	<0.0001 *
B—Surfactant (%)	481.19	<0.0001 *	39.21	0.0015 *
C—Ethanol (%)	841.32	<0.0001 *	3120.41	<0.0001 *
Lack of Fit	2.20	0.3272	0.4580	0.7401
R^2^ analysis				
R²	0.9971		0.9991	
Adjusted R²	0.9920		0.9976	
Predicted R²	0.9632		0.9933	
Adequate Precision	48.3566		96.87	
%CV	0.95		1.05	
SD	1.54		5.87	

* Significant.

**Table 3:** Box–Behnken design of erythromycin loaded transethosomes optimization.

Independent Variable	Symbol	Level of Variation		
		−1	0	+1
Phospholipid concentration (%)	A	2	3	4
Surfactant concentration (%)	B	10	20	30
Ethanol concentration (%)	C	20	35	50
**Independent Variable**	**Unit**		**Constraints**	
Entrapment efficiency (Y_1_)	%		Maximize	
Cumulative amount of drug permeated after 6 h, Q6h (Y_2_)	µg/cm^2^		Maximize	

## Data Availability

Not applicable.
